# Transcriptional alterations in *Caenorhabditis elegans* following exposure to an anthelmintic fraction of the plant *Picria fel-terrae* Lour.

**DOI:** 10.1186/s13071-019-3429-4

**Published:** 2019-04-25

**Authors:** Rasika Kumarasingha, Neil D. Young, Tiong-Chia Yeo, Diana S. L. Lim, Chu-Lee Tu, Enzo A. Palombo, Jillian M. Shaw, Robin B. Gasser, Peter R. Boag

**Affiliations:** 1 Development and Stem Cells Program, Monash Biomedicine Discovery Institute, Clayton, Victoria 3800 Australia; 2 Department of Biochemistry and Molecular Biology, Monash University, Clayton, Victoria 3800 Australia; 3 Faculty of Veterinary and Agricultural Sciences, The University of Melbourne, Parkville, Victoria 3010 Australia; 4 Sarawak Biodiversity Centre, KM 20 Jalan Borneo Heights, Semengoh, Locked Bag 3032, 93990 Kuching, Sarawak Malaysia; 5 Department of Chemistry and Biotechnology, Faculty of Science, Engineering and Technology, Swinburne University of Technology, Hawthorn, Victoria 3122 Australia; 6 Department of Health and Medical Sciences, Faculty of Health, Arts and Design, Swinburne University of Technology, Hawthorn, Victoria 3122 Australia

**Keywords:** Medicinal plant extracts, *Picria fel-terrae* Lour., Anthelmintic activity, *Caenorhabditis elegans*, RNA sequencing, Bioinformatics

## Abstract

**Background:**

Natural compounds from plants are known to provide a source of anthelmintic molecules. In previous studies, we have shown that plant extracts from the plant *Picria fel-terrae* Lour. and particular fractions thereof have activity against the free-living nematode *Caenorhabditis elegans*, causing quite pronounced stress responses in this nematode. We have also shown that a fraction, designated *Pf-fraction 5*, derived from this plant has a substantial adverse effect on this worm; however, nothing is known about the molecular processes affected in the worm. In the present study, we explored this aspect.

**Results:**

Key biological processes linked to upregulated genes (*n* = 214) included ‘response to endoplasmic reticulum stress’ and ‘lipid metabolism’, and processes representing downregulated genes (*n* = 357) included ‘DNA-conformation change’ and ‘cellular lipid metabolism’.

**Conclusions:**

Exposure of *C. elegans* to *Pf-fraction 5* induces significant changes in the transcriptome. Gene ontology analysis suggests that *Pf-fraction 5* induces endoplasmic reticulum and mitochondrial stress, and the changes in gene expression are either a direct or indirect consequence of this. Further work is required to assess specific responses to sub-fractions of *Pf-fraction 5* in time-course experiments in *C. elegans*, to define the chemical(s) with potent anthelmintic properties, to attempt to unravel their mode(s) of action and to assess their selectivity against nematodes.

**Electronic supplementary material:**

The online version of this article (10.1186/s13071-019-3429-4) contains supplementary material, which is available to authorized users.

## Background

Parasitic nematodes cause substantial mortality and morbidity in humans and animals worldwide. For instance, gastrointestinal nematodes of livestock affect hundreds of millions of small ruminants (including goats and sheep) and cause substantial losses to the livestock industry estimated at tens of billions of dollars per annum worldwide [1]. The control of these nematodes has predominantly relied on the use of anthelmintic drugs, but the excessive and uncontrolled use of such drugs has led to widespread resistance in these nematodes to most anthelmintic classes [2], thus seriously compromising the control of parasites in many countries. Although the development of the compounds monepantel and derquantel (2-deoxy-paraherquamide) [3–5] has provided some optimism about commercialising new classes of nematocides, success in bringing new anthelmintics to market has been limited over the last years.

Natural compounds from plants provide a unique opportunity in the search for new, effective and safe anthelmintics [6–10]. In China, for example, plant-derived medicines have been used for centuries to treat many disease conditions, including parasitoses, in humans [11, 12] and other animals [13–15]. It is possible that some natural medicines act on pathways in nematodes that differ from targets of currently used anthelmintic drugs [16] and, therefore, might be able to kill nematodes that are resistant to one or more commercial anthelmintics. However, for most natural compounds, there has been limited systematic, scientific evaluations of efficacy, mode of action and identity of their active component(s), and no plant-derived anthelmintic has yet prospered commercially [17].

Recently, we evaluated eight plant extracts from different parts of *Picria fel-terrae* Lour.*, Lansium domesticum*, *Linariantha bicolor* and *Tetracera akara* for nematocidal activity in seven strains of the free-living nematode *Caenorhabditis elegans*, and characterised stress responses induced by these extracts [18]. Five of the eight extracts from plants that have been used by Malaysian healers to treat worm infections and gastrointestinal disorders in humans [19, 20] had significant nematocidal activity against both adult and larval stages of *C. elegans* [18]. The most effective extracts were derived from *P. fel-terrae*, and triggered stress response pathways that were distinct from two commercially available anthelmintics (levamisole and doramectin) [18]. In a subsequent study [21], we showed that extracts from *P. fel-terrae* Lour. had a significant inhibitory effect on the motility and development of *H. contortus* larvae. A subsequent study, conducted to fractionate the plant extract of *P. fel-terrae* Lour., identified a fraction, designated *Pf-fraction 5*, with substantial anthelmintic activity [22]. In spite of this information, it is not known how *P. fel-terrae* plant extracts or *Pf-fraction 5* affect(s) the nematode. As transcriptomic investigations can provide a global snapshot of molecular alterations in an organism and clues about modes of action [23], here we elected to logically extend previous work to explore the transcriptional differences in *C. elegans* exposed to *Pf-fraction 5* compared with untreated controls, and assessed which biological pathways are affected by this plant extract fraction.

## Methods

### Maintenance of *C. elegans*

*Caenorhabditis elegans* strain N2 Bristol was used and maintained using established methods [24] and were maintained on Nematode Growth Medium (NGM; 3% bacto-agar, 86 mM NaCl, 42 mM Na_2_HPO_4_, 22 mM KH_2_PO_4_ and 1 mM MgSO_4_) on a lawn of *Escherichia coli* (strain OP50) at 20 °C. Synchronized populations of *C. elegans* were obtained by a modified alkaline bleaching method [25]. Eggs and egg-laying adults were washed briefly in M9 buffer (86 mM NaCl, 42 mM Na_2_HPO_4_, 22 mM KH_2_PO_4_ and 1 mM MgSO_4_) and then incubated in bleaching solution (4 ml of commercial bleach, 1 ml of 1 M NaOH and 9 ml of H_2_O) for 3.5 min. Eggs were pelleted by centrifugation (1000×*g*) and washed three times in M9 and then incubated at 20 °C (rotating) for at least 20 h. To obtain young adults, L1s were transferred to NGM plates and maintained at 20 °C for 48 h. Young adults were isolated, washed four times in M9 before being used in experiments.

### Preparation of *Pf-fraction 5*

*Picria fel-terrae* Lour. whole-plant extract was prepared at the Sarawak Biodiversity Centre, Kuching, Malaysia [18]. A high quality solid phase extraction strata C18-E column (Silica-based sorbent, Phenomenex, Torrance, USA) was used to fractionate the extract [18]. To do this, 100 mg of dried extract was dissolved in 1 ml of absolute ethanol and then diluted with 19 ml of H_2_O. The column was washed with 20 ml of 100% acetonitrile (ACN, Sigma, Castle Hill, Australia) and conditioned using 20 ml of H_2_O. The dissolved plant extract was then loaded on to the C18-E column and the eluate collected. This step was followed by successive elutions with 20 ml of different percentages of ACN (10%, 25%, 40%, 55%, 70%, 85% and 100%) from the column and collecting eluted fractions at the stationary phase. *Pf-fraction 5* collected using 40% ACN was dried by rotary evaporation at 4 °C, and then reconstituted in 1 ml of absolute ethanol. For treatment of *C. elegans*, a working stock of *Pf-fraction 5* was prepared by diluting a 10 µl aliquot into 990 µl of M9 (1000-fold dilution) [22].

### Treatment of *C. elegans* with *Pf-fraction 5*

Paired samples of young adults (*n* = 1000) were incubated in diluted *Pf-fraction 5* (‘treated *C. elegans*’) or M9 containing 1% ethanol (‘untreated controls’) at 20 °C for 12 h. The *C. elegans* exposed to *Pf-fraction 5* under this condition exhibited a stress phenotype, but did not immediately die [18]. Following incubation for 12 h, treated and untreated *C. elegans* were separately harvested, snap frozen in liquid nitrogen and stored at − 80 °C for subsequent molecular investigation.

### RNA extraction

RNA was extracted from two independent biological replicates of ‘treated’ and ‘untreated’ *C. elegans* using the TRIzol® reagent (Invitrogen, Waltham, USA) according to the manufacturer’s instructions. To facilitate total RNA precipitation, 2 μl GlycoBlue (Ambion, Austin, USA) co-precipitant were added. Total RNA was suspended in 90 μl of RNase-free H_2_O and treated with *DN*ase (TURBO DNA-free kit, Life Technologies, Carlsbad, USA). RNA integrity was assessed using a Bioanalyzer (Agilent, Santa Clara, USA), and RNA samples were stored at − 80 °C for subsequent sequencing.

### Illumina RNA-Seq sequencing and bioinformatic analyses

Pairs of mRNA libraries of *Pf-fraction 5*-‘treated *C. elegans*’ and ‘untreated *C. elegans*’ were constructed (TruSeq® RNA Sample Preparation v.2, Illumina, San Diego, USA) and sequenced (HiSeq™ 2500 sequencing platform, Illumina) according to the manufacturer’s protocols. Each of the four libraries contained ~ 25 million reads (Additional file 1: Table S1).

Reads were processed in the FASTQ format [26]. Nucleotide sequences of a Phred quality of < 25, adapters and reads of < 50 nucleotides (nt) in length were removed using the program Trimmomatic [27]. The quality of reads from individual libraries was then assessed using FastQC (http://www.bioinformatics.babraham.ac.uk/projects/fastqc/). For each replicate, high quality paired reads were mapped to the protein coding regions within the *C. elegans* reference genome (v.WBcel235; INSDC Assembly GCA_000002985.3) using RSEM v.1.2.21 [28]. Expected counts determined for each gene were submitted to EBSeq (v.1.1.5) [29] employing median normalisation and a false discovery rate (FDR) of 0.05, to determine the posterior probability of each gene being differentially transcribed by more than a 2-fold change in gene transcription between ‘treated’ and ‘untreated’ *C. elegans*. Enriched GO terms for gene sets recorded to be differentially transcribed were predicted using BINGO [30], and the hypergeometric test and Benjamini & Hochberg False Discovery Rate (FDR) correction (adjusted *P* ≤ 0.05) applied.

Representative enriched gene ontology (GO) terms were inferred using the program REVIGO [31]. Each protein-encoding gene was assigned to a Kyoto Encyclopedia of Genes and Genomes (KEGG) orthologous gene (KO) group using established methods [32]. Individual genes linked to one or more KO terms were assigned to known protein families and biological pathways using the KEGG BRITE and KEGG PATHWAY hierarchies employing custom python scripts. Enriched protein families and biological pathways represented by ≥ 5 genes were defined using the Fisher’s exact test, employing a custom script and linking data to KEGG biological pathway BRITE hierarchy.

## Results and discussion

We compared transcription in *Pf-fraction 5*-treated *C. elegans* with untreated controls, and established the molecular pathways affected by treatment with this fraction of whole-plant extract from *P. fel-terrae*. Four cDNA sequencing libraries (two technical replicates for each ‘treated’ and ‘untreated’ *C. elegans*) yielded 99,732,338 high quality reads (49,724,404 for ‘treated’ 50,007,934 for ‘untreated’ *C. elegans*) (Additional file 1: Table S1). Raw RNA-Seq read data are available for download from the NCBI sequence read archive (BioProject ID: PRJNA301756). More than 91% of high quality RNA-Seq reads from each library mapped to the *C. elegans* reference genome (range: 91.8–92.4%) (Additional file 1: Table S1). Compared with the ‘untreated’ group, we identified 214 upregulated and 357 downregulated protein-coding genes (≥ 0.95 probability of differential transcription and ≥ 2 fold-change in transcription in ‘treated’ *C. elegans* (Additional file 2: Figure S1, Additional file 3: Table S2). Protein families enriched in the upregulated gene set were assigned to seven KEGG BRITE protein families, and included transporters, transcription factors, spliceosomes, nuclear receptors, lipid biosynthesis proteins, enzymes, chaperones and protein catalysts (Additional file 4: Table S3). Pathways enriched in this upregulated set of 214 genes associated with environmental information processing (including signal transduction and MAPK signalling), genetic information processing (including folding, sorting and degradation and protein processing in the endoplasmic reticulum) and metabolism (including lipid metabolism and fatty acid degradation) (Additional file 5: Table S4).

Protein families enriched in the set of the 357 downregulated genes in ‘treated’ *C. elegans* were assigned to eight KEGG BRITE protein families, namely transporters, peptidases, mitochondrial biogenesis proteins, lectins, glycosyltransferases, enzymes, oxidoreductases and hydrolases (Additional file 4: Table S3). Pathways enriched in the downregulated gene set associated with cellular processes (including transport and catabolism in lysosomes and peroxisomes) and metabolism of carbohydrates (ascorbate and aldarate metabolism, pentose and glucuronate interconversions and starch and sucrose metabolism), lipids (alpha-linolenic acid metabolism, biosynthesis of unsaturated fatty acids, fatty acid degradation, glycerophospholipid metabolism, steroid hormone biosynthesis) and xenobiotics (aminobenzoate degradation, drug metabolism by cytochrome P450 and other enzymes).

**Fig. 1:**
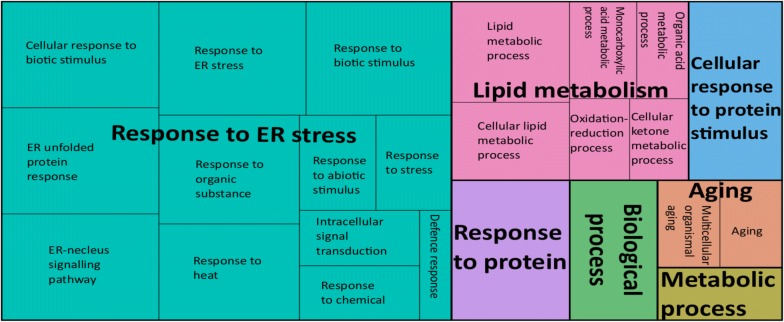
A gene ontology (GO) map of 214 upregulated genes in *C. elegans* in response to treatment with *Pf-fraction 5* and linked to conserved biological processes. Individual rectangles represent single gene ontology terms, which are arranged into ‘superclusters’ (related terms), shown in different colors. The size of each rectangle reflects the frequency of individual GO terms representing the upregulated genes

**Fig. 2:**
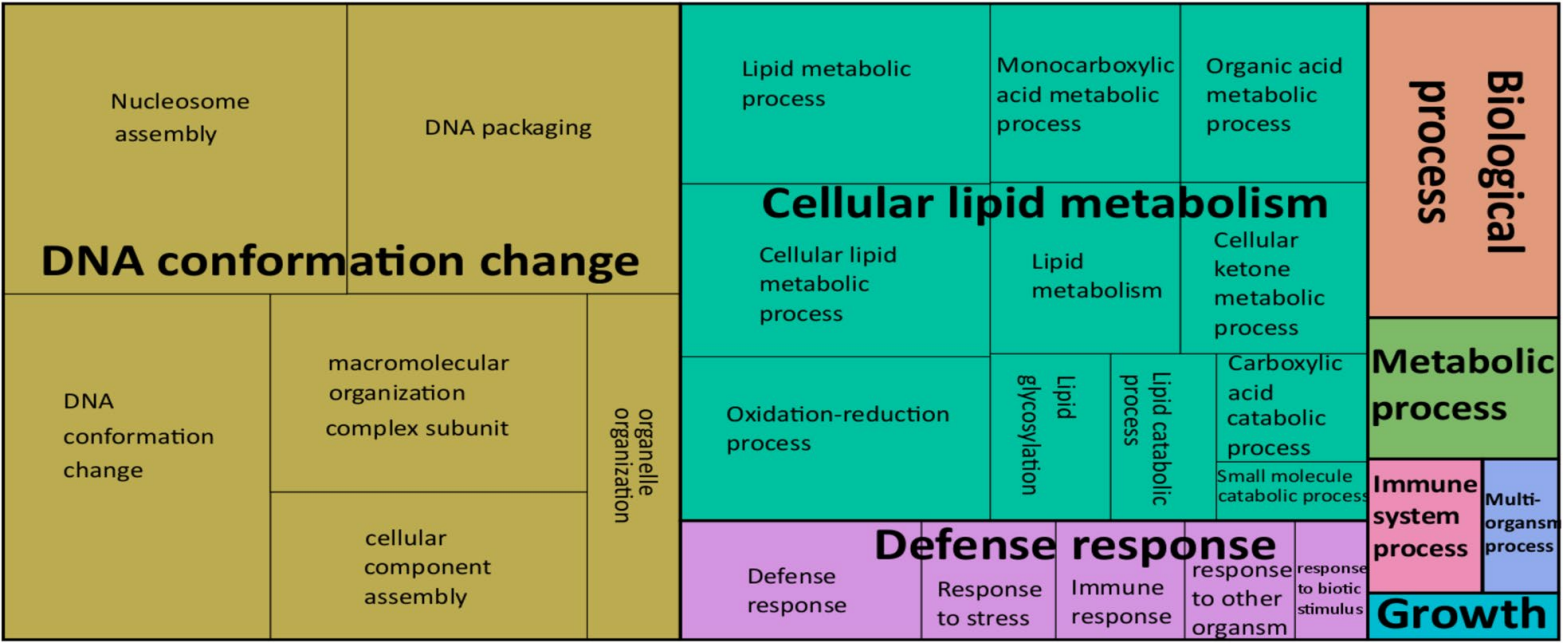
A gene ontology (GO) map of 357 downregulated genes in *C. elegans* in response to treatment with *Pf-fraction* 5 and linked to conserved biological processes. Individual rectangles represent single gene ontology terms, which are arranged into ‘superclusters’ (related terms), shown in different colors. The size of each rectangle reflects the frequency of individual GO terms representing the downregulated genes

We identified seven and eight GO terms that were significantly enriched for up- and downregulated gene sets, respectively (Additional file 6: Table S5). The key biological processes representing upregulated genes included ‘response to endoplasmic reticulum (ER) stress’ and ‘lipid metabolism’ (Fig. 1), and processes representing the downregulated genes included ‘DNA-conformation change’ and ‘cellular lipid metabolism’ (Fig. 2).

### Endoplasmic reticulum (ER) stress

Thirteen genes relating to ER structure and function, including *abu-1*, *abu-7*, *abu-15*, *pqn-2* and 5 *hsp-16* genes, were upregulated in *Pf-fraction 5*-treated *C. elegans* (Additional file 6: Table S5). The *abu-1* and *abu-7* genes encode ER transmembrane proteins, and their upregulation is induced by the presence of unfolded protein in ER [33, 34]. The five upregulated *hsp-16* genes are predicted to function as passive ligands to temporarily prevent unfolded proteins from aggregating [35, 36]. This information indicates that the exposure of *C. elegans* to *Pf-fraction 5* leads to stress and dysfunction in ER. How this ER stress is induced is unclear, as we studied transcription 12 h following exposure to *Pf-fraction 5*. The ER stress might be a direct result of exposure to *Pf-fraction 5*, but could also be a downstream consequence of this exposure *via* a presently unknown mechanism or process.

Cells respond to stress conditions by inducing a stress response, and associated genes are usually conserved among organisms, including nematodes [37]. An essential component of the stress response is an upregulation of heat-shock proteins (HSPs). HSPs act as molecular chaperones and bind to non-native conformations of proteins that persist upon cell stress [37]. These interactions prevent misfolding, aggregation and/or premature clearance of proteins, and enable cells to restore the necessary folding [38, 39]. In a healthy cellular environment, elevated levels of HSPs are adequate to protect cells from stress situations [40, 41]. However, if this mechanism fails to adequately respond to proteo-toxic imbalances, unfolded/misfolded proteins are accumulated [42]. The present transcriptomic analysis is consistent with an increase of unfolded proteins in *Pf-fraction 5* treated *C. elegans*. The upregulation of the HSPs is usually associated with a downregulation of genes linked to normal cellular functions [40, 41], which might be a reason for the downregulation of many genes involved in a wide range of biological processes, as seen here (Fig. 2).

The *hsp-70* multi-gene family of *C. elegans* has unique characteristics, and 12 genes in this family have been studied [43–45]. The proteins encoded by these genes function as molecular chaperones for unfolded proteins [37, 46]. One subfamily consists of an HSP-70 that can be translocated into ER, and another subfamily consists of a protein that can be translocated into the mitochondria [44]. In the present study, the transcription of three *hsp-70* genes was upregulated, indicating the presence of unfolded proteins and a cellular response to the stressor (*Pf-fraction 5*). ER stress can be induced by the accumulation of unfolded protein aggregates (unfolded protein response, UPR), which leads to cell death by apoptosis [47–49]. ER is the principal organelle of the cell for lipid synthesis, protein folding and protein maturation [47–49], and is the major signal transducing organelle that senses and responds to changes of protein homeostasis [47–49]. Therefore, the normal function of ER is essential for cell functions and, consequently, for the survival of the organism. If ER stress is not corrected in *C. elegans*, cells are damaged and removed by apoptosis [47–49]. This might explain the molecular and morphological changes seen in *C. elegans* exposed to *Pf-fraction 5*, which eventually led to the death of treated *C. elegans* after 48 h of exposure.

The mitochondrion is the powerhouse of the cell, producing ATP by oxidative phosphorylation. The function of the mitochondria can be challenged by exposure of the cells to toxins as well as pathogens. If the mitochondria are damaged, electrons can escape from the sites in the respiratory chain and react with oxygen to form reactive oxygen species (ROS), which are well known for creating oxidative stress [50, 51]. The constant exposure to *Pf-fraction 5* may have overwhelmed the cell’s ability to detoxify the active agent(s) and induced ER stress. In parallel, detoxification of active components within *Pf-fraction 5* might have stressed mitochondria and ultimately led to their dysfunction or destruction. This proposal could explain the downregulation of the fatty acid oxidation and energy production processes of mitochondria. Alternatively, the observation that components of the unfolded protein response pathway are induced by *Pf-fraction 5* might indicate that fatty acid metabolism and mitochondrial function are impacted by defective protein folding and/or importation into the mitochondria. These aspects require investigation.

The ER protein folding mechanism and its chaperones have been considered as new drug targets [42]. The *Plasmodium* mitochondrion is a validated drug target [52]; for example, the inner membrane electron transport chain is the target of the antimalarial drug atovaquone [53]. Moreover, the potential of targeting *Trypanosoma cruzi* mitochondria as a new drug target has been shown [54], and the cytochrome 450 family is being studied as an anti-cancer drug target [55]. This information suggests that targeting ER and/or mitochondria might have potential for designing or discovering a new nematocide, but further work is required to establish whether this might apply to *Pf-fraction 5*.

Both ER stress and oxidative stress often induce apoptosis (programmed cell death) in *C. elegans*. This is characterised by an upregulation of *egl-1*, *ced-3*, *ced-4* and *ced-9* in *C. elegans* germ cells or developing cells. However, such stress can also be activated to maintain homeostasis in disease conditions [56]. In the present study, apoptosis marker genes were not significantly upregulated in *C. elegans* following treatment with *Pf-fraction 5*. However, given the pattern of the ER stress, such an upregulation might be detected at a later stage if deep sequencing were performed in a time-course experiment. Furthermore, as the biochemical pathway(s) used by parasitic helminths to bio-transform commercial anthelmintics are often unknown [57], detailed investigations of detoxification mechanisms are warranted.

### Lipid metabolism and downregulation of β-oxidation in mitochondria

As this study was performed in *C. elegans* with no access to a food source, a starvation response was expected. However, the *nhr-49* gene, which is essential for the activation and repression of starvation genes [58, 59], was not differentially abundant between *Pf-fraction 5*-‘treated’ and ‘untreated’ *C. elegans*, assuming that both groups of *C. elegans* respond to starvation in the same way. Therefore, theoretically, starvation response genes should not be differentially transcribed, unless the chemicals within *Pf-fraction 5* affect the energy metabolism of the treated *C. elegans*.

**Fig. 3:**
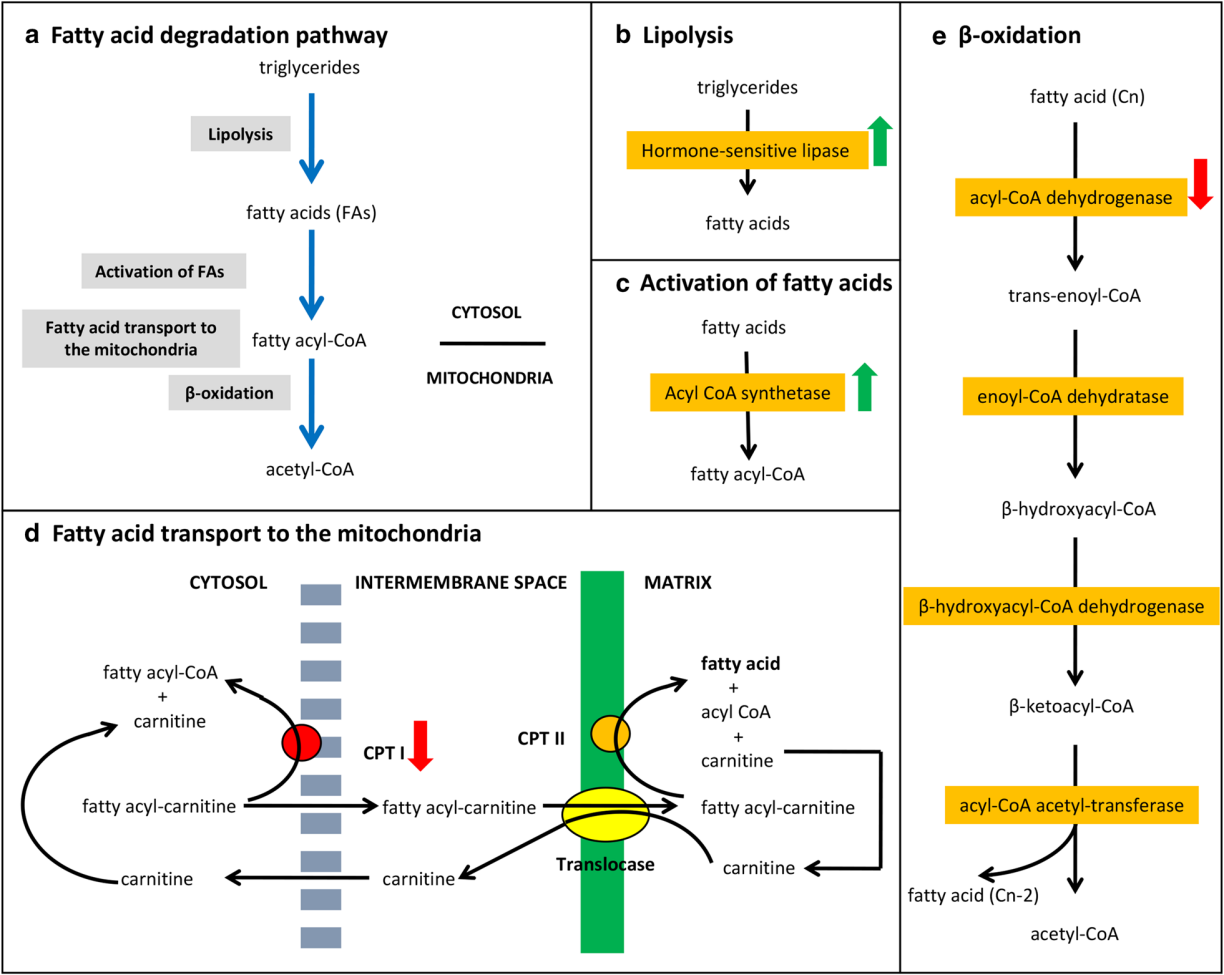
Fatty acid degradation processes/pathways in *C. elegans*. **a** Schematic diagram of fatty acid degradation process. **b** Lipolysis. **c** Activation of fatty acids. **d** Fatty acid transport to the mitochondria. **e** β-oxidation (adapted from Nelson and Cox [60]). Up-arrow indicates increase transcript abundance, down-arrow indicates decrease transcript abundance

The genes *hosl-1* and *acs-2*, which encode key enzymes in β-oxidation pathway, were upregulated. In fatty acid oxidation pathway, triglycerides are first broken-down to fatty acids (lipolysis) [60] and the long chain fatty acids are converted to their fatty acid-CoA derivative (activation) in the cytosol (Fig. 3a–c). The gene *hosl-1* encodes the *C. elegans* ortholog of hormone-sensitive lipase [61], which catalyzes the first rate-limiting step of triglyceride hydrolysis [60]. The *acs-2* gene encodes an acyl-CoA synthetase, which is predicted to catalyze the conversion of a fatty acid to fatty acyl-CoA for subsequent β-oxidation in the mitochondria [58, 60]. An upregulation of these enzymes suggests that the fatty acids stores within *C. elegans* are being used for energy production.

For β-oxidation, long chain fatty acids need to be transported across the inner mitochondrial membrane (impermeable to CoA) to the mitochondrial matrix. This is mediated by carnitine palmitoyltransferase I (CPT-1), which is located on the outer membrane of the mitochondria and replaces CoA from fatty acid-CoA with carnitine to form fatty acyl carnitine. The fatty acyl carnitine can then be translocated through the inner mitochondrial membrane. Then, the CPT-2 transports the fatty acids across the mitochondrial inner membrane to the mitochondria matrix where β-oxidation occurs. In this study, *cpt-1* was downregulated (Fig. 3d), but *cpt-2* was not affected. We hypothesise that *Pf-fraction 5-*treated *C. elegans*, despite having increased levels of lipolysis, would have reduced levels of β-oxidation as the long chain fatty acids would not have been efficiently transported into the mitochondrial matrix for β-oxidation.

The genes *acdh-1* and *acdh-2* encode short chain acyl-CoA dehydrogenases, which catalyze the first step of fatty acid beta-oxidation in mitochondria (Fig. 3e) [62]. While the transcription of genes *acdh-1* and *acdh-2* were downregulated in response to *Pf-fraction* 5, other genes associated with β-oxidation were not affected. These findings suggest that constituents of *Pf-fraction 5* specifically target two major rate limiting steps in fatty acid degradation in *C. elegans*.

Nematodes, including *C. elegans*, are highly dependent on lipids for energy storage and energy metabolism [63], with the intestine being a major site of lipid storage and metabolism [64, 65]. Mitochondria and the peroxisomes are the two sites of fatty acid oxidation in *C. elegans*, and most peroxisomes are in the intestine [66]. The genes *elo-1*, *elo-5* and *elo-6* encode enzymes that have fatty acid elongation activities, and are involved in the synthesis of polyunsaturated and branched fatty acids [67]. In this study, the *elo-5* gene was upregulated in response to *Pf-fraction 5*. All 3 Δ-9-desaturases in *C. elegans*, encoded by *fat-5*, *fat-6* and *fat-7*, respectively, are expressed exclusively in the intestine [68]. Collectively, the present data suggest that lipid homeostasis is disrupted in *C. elegans* exposed to *Pf-fraction 5*. Interestingly, *C. elegans* exposed to ivermectin also displayed a substantial change in gene transcription associated with lipid mobilisation and fatty acid metabolism [69]. Although the mechanism of these changes might be distinct from those reported here, it does highlight the sensitivity of exogenous influences on metabolic processes.

## Conclusions

In the present study, we provide evidence that exposure of *C. elegans* to *Pf-fraction 5* elicits substantial changes in transcription. Genes associated with endoplasmic reticulum and mitochondrial stresses are significantly affected, which is in agreement with our previous work [18]. Although the molecular events that lead to these changes are presently unclear, they likely represent a complex combination of “secondary” responses in *C. elegans* to exposure to *Pf-fraction 5*. Significant further transcriptomic and biochemical work is required to understand how *Pf-fraction 5* kills *C. elegans*. On the other hand, as this fraction likely contains numerous chemical compounds [22], identifying the specific responses induced by particular active constituents causing anthelmintic activity might be a challenging task. Nonetheless, sub-fractionation of *Pf-fraction 5* is required to assess specific responses, and work is needed to dissect the modes of action of individual components or molecules within this anthelmintic fraction of the plant *Picria fel-terrae* Lour.

## Additional files

**Additional file 1: Table S1.** Summary of sequence libraries and RNAseq data aligned to the *C. elegans* mRNA.

**Additional file 2: Figure S1.** Differential transcription of protein-encoding genes in *C. elegans* upon exposure to an anthelmintic fraction of the plant *Picria fel-terrae* Lour. compared with untreated *C. elegans*. Genes significantly differentially transcribed at > 2-fold change and a posterior probability (*PP*) of > 0.95 (red dots); at > 2-fold change and a *PP* of < 0.95 (grey dots); at < 2-fold change and a *PP* of < 0.95 (black dots).

**Additional file 3: Table S2.** List of differentially transcribed genes using only *n* = 2 and removing outliers (F5.2 and M9.3), including expected counts for biological replicates and using a cutoff of FDR 0.05 and a 2-fold change in transcription.

**Additional file 4: Table S3.** KEGG BRITE terms predicted to be significantly enriched in gene sets up and downregulated in *C. elegans* treated with fraction 5 (F5).

**Additional file 5: Table S4.** KEGG pathway terms predicted to be significantly enriched in gene sets up and downregulated in *C. elegans* treated with fraction 5 (F5).

**Additional file 6: Table S5.** Gene ontology terms predicted to be significantly enriched in gene sets up and downregulated in *C. elegans* treated with fraction 5 (F5).

## Abbreviations

ER: endoplasmic reticulum
GO: gene ontology
KEGG: Kyoto Encyclopedia of Genes and Genome
KO: KEGG Orthology
NGM: Nematode Growth Medium
MAPK: mitogen-activated protein kinase

## References

[1] Roeber F, Jex AR, Gasser RB (2013). Impact of gastrointestinal parasitic nematodes of sheep, and the role of advanced molecular tools for exploring epidemiology and drug resistance—an Australian perspective. Parasite Vectors.

[2] Kotze AC, Prichard RK (2016). Anthelmintic resistance in *Haemonchus contortus*: history, mechanisms and diagnosis. Adv Parasitol.

[3] Kaminsky R, Ducray P, Jung M, Clover R, Rufener L, Bouvier J (2008). A new class of anthelmintics effective against drug-resistant nematodes. Nature.

[4] Prichard RK, Geary TG (2008). Drug discovery: fresh hope to can the worms. Nature.

[5] Epe C, Kaminsky R (2013). New advancement in anthelmintic drugs in veterinary medicine. Trends Parasitol.

[6] Hammond JA, Fielding D, Bishop SC (1997). Prospects for plant anthelmintics in tropical veterinary medicine. Vet Res Commun..

[7] Fajimi AK, Taiwo AA (2005). Herbal remedies in animal parasitic diseases in Nigeria: a review. Afr J Biotechnol.

[8] Koné WM, Atindehou KK, Dossahoua T, Betschart B (2005). Anthelmintic activity of medicinal plants used in northern Côte d’Ivoire against intestinal helminthiasis. Pharm Biol..

[9] Harvey AL, Edrada-Ebel R, Quinn RJ (2015). The re-emergence of natural products for drug discovery in the genomics era. Nat Rev Drug Discov..

[10] Boufridi A, Quinn RJ (2018). Harnessing the properties of natural products. Annu Rev Pharmacol Toxicol..

[11] Liu ZL, Liu JP, Zhang AL, Wu Q, Ruan Y, Lewith G, Visconte D (2011). Chinese herbal medicines for hypercholesterolemia. Cochrane Database Syst Rev..

[12] Xu HB, Jiang RH, Chen XZ, Li L (2012). Chinese herbal medicine in treatment of diabetic peripheral neuropathy: a systematic review and meta-analysis. J Ethnopharmacol..

[13] Hon KLE, Ma KC, Wong Y, Leung TF, Fok TF (2005). A survey of traditional Chinese medicine use in children with atopic dermatitis attending a paediatric dermatology clinic. J Dermatol Treat.

[14] Li ZH, Wan JY, Wang GQ, Zhao FG, Wen JH (2013). Identification of compounds from *Paris polyphylla* (ChongLou) active against *Dactylogyrus intermedius*. Parasitology..

[15] Zhu L, Dai JL, Yang L, Qiu J (2013). *In vitro* ovicidal and larvicidal activity of the essential oil of *Artemisia lancea* against *Haemonchus contortus* (Strongylida). Vet Parasitol..

[16] Hrckova G, Velebny S. Parasitic helminths of humans and animals: health impact and control. In: Pharmacological potential of selected natural compounds in the control of parasitic diseases. Vienna: Springer; 2013. p. 29–99.

[17] Behnke JM, Buttle DJ, Stepek G, Lowe A, Duce IR (2008). Developing novel anthelmintics from plant cysteine proteinases. Parasite Vectors..

[18] Kumarasingha R, Palombo EA, Bhave M, Yeo TC, Lim DSL, Tu CL (2014). Enhancing a search for traditional medicinal plants with anthelmintic action by using wild type and stress reporter *Caenorhabditis elegans* strains as screening tools. Int J Parasitol..

[19] Christensen H (2002). Ethnobotany of the Iban & the Kelabit.

[20] Chai PPK. Medicinal plants of Sarawak. Kuching, Sarawak, Malaysia: Paul Chai P.K.; 2006.

[21] Kumarasingha R, Preston S, Yeo TC, Lim DSL, Tu CL, Palombo EA (2016). Anthelmintic activity of selected ethno-medicinal plant extracts on parasitic stages of *Haemonchus contortus*. Parasite Vectors..

[22] Kumarasingha R, Karpe AV, Preston S, Yeo TC, Lim DSL, Tu CL (2016). Metabolic profiling and *in vitro* assessment of anthelmintic fractions of *Picria fel-terrae* Lour. Int J Parasitol Drugs Drug Resist..

[23] Preston S, Korhonen PK, Mouchiroud L, Cornaglia M, McGee SL, Young ND (2017). Deguelin exerts potent nematocidal activity *via* the mitochondrial respiratory chain. FASEB J..

[24] Brenner S (1974). The genetics of *Caenorhabditis elegans*. Genetics.

[25] Lenaerts I, Walker GA, Van HL, Gems D, Vanfleteren JR (2008). Dietary restriction of *Caenorhabditis elegans* by axenic culture reflects nutritional requirement for constituents provided by metabolically active microbes. J Gerontol A Biol Sci Med Sci..

[26] Cock P, Fields C, Goto N, Heuer M, Rice P (2010). The Sanger FASTQ file format for sequences with quality scores, and the Solexa/Illumina FASTQ variants. Nucleic Acids Res..

[27] Bolger AM, Lohse M, Usadel B (2014). Trimmomatic: a flexible trimmer for Illumina sequence data. Bioinformatics..

[28] Li B, Dewey CN (2011). RSEM: accurate transcript quantification from RNA-Seq data with or without a reference genome. BMC Bioinform..

[29] Leng N, Dawson JA, Thomson JA, Ruotti V, Rissman AI, Smits BMG (2013). EBSeq: an empirical Bayes hierarchical model for inference in RNA-seq experiments. Bioinformatics..

[30] Maere S, Heymans K, Kuiper M (2006). BiNGO: a Cytoscape plugin to assess overrepresentation of gene ontology categories in biological networks. Bioinformatics..

[31] Supek F, Bošnjak M, Škunca N, Šmuc T (2011). Revigo summarizes and visualizes long lists of gene ontology terms. PLoS ONE..

[32] Xie C, Mao X, Huang J, Ding Y, Wu J, Dong S (2011). KOBAS 2.0: a web server for annotation and identification of enriched pathways and diseases. Nucl Acids Res..

[33] Harding HP, Calfon M, Urano F, Novoa I, Ron D (2002). Transcriptional and translational control in the mammalian unfolded protein response. Ann Rev Cell Dev Biol..

[34] Urano F, Calfon M, Yoneda T, Yun C, Kiraly M, Clark SG (2002). A survival pathway for *Caenorhabditis elegans* with a blocked unfolded protein response. J Cell Biol..

[35] Jones D, Dixon DK, Graham RW, Candido EP (1989). Differential regulation of closely related members of the *hsp*16 gene family in *Caenorhabditis elegans*. DNA..

[36] Mogk A, Schlieker C, Friedrich KL, Schönfeld HJ, Vierling E, Bukau B (2003). Refolding of substrates bound to small HSPs relies on a disaggregation reaction mediated most efficiently by ClpB/DnaK. J Biol Chem..

[37] Prahlad V, Morimoto PI (2009). Integrating the stress response: lessons for neurodegenerative diseases from *C. elegans*. Trends Cell Biol..

[38] Hartl FU, Hlodan R, Langer T (1994). Molecular chaperones in protein folding: the art of avoiding sticky situations. Trends Biochem Sci..

[39] Bukau B, Weissman J, Horwich A (2006). Molecular chaperones and protein quality control. Cell..

[40] Lindquist S, Craig EA (1988). The heat-shock proteins. Annu Rev Genet..

[41] Morimoto RI (2008). Proteotoxic stress and inducible chaperone networks in neurodegenerative disease and aging. Genes Dev..

[42] Mclaughlin M, Vandenbroeck K (2011). The endoplasmic reticulum protein folding factory and its chaperones: new targets for drug discovery?. Br J Pharmacol..

[43] Heschl MFP, Baillie DL (1989). Characterization of the *hsp*70 multigene family of *Caenorhabditis elegans*. DNA..

[44] Heschl MFP, Baillie DL (1990). The HSP70 multigene family of *Caenorhabditis elegans*. Comp Biochem Physiol B..

[45] Morley JF, Morimoto RI (2004). Regulation of longevity in *Caenorhabditis elegans* by heat shock factor and molecular chaperones. Mol Biol Cell..

[46] Ellis RJ (1997). Molecular chaperones: avoiding the crowd. Curr Biol..

[47] Kapulkin V, Hiester BG, Link CD (2005). Compensatory regulation among ER chaperones in *Caenorhabditis elegans*. FEBS Lett..

[48] Ient B, Edwards R, Mould R, Hannah M, Holden-Dye L, O’Connor V (2012). HSP-4 endoplasmic reticulum (ER) stress pathway is not activated in a *Caenorhabditis elegans* model of ethanol intoxication and withdrawal. Invertebr Neurosci.

[49] Zhang H, Zhou Q, Yang Y, Chen X, Yan B, Du A (2013). Characterization of heat shock protein 70 gene from *Haemonchus contortus* and its expression and promoter analysis in *Caenorhabditis elegans*. Parasitology..

[50] Brookes PS, Levonen AL, Shiva S, Sarti P, Darley-Usmar VM (2002). Mitochondria: regulators of signal transduction by reactive oxygen and nitrogen species. Free Radic Biol Med..

[51] Fernández-Checa JC (2003). Redox regulation and signaling lipids in mitochondrial apoptosis. Biochem Biophys Res Commun..

[52] Van Dooren GG, Stimmler LM, McFadden GI (2006). Metabolic maps and functions of the *Plasmodium* mitochondrion. FEMS Microbiol Rev..

[53] Fry M, Pudney M (1992). Site of action of the antimalarial hydroxynaphthoquinone, 2-[trans-4-(4′-chlorophenyl) cyclohexyl]-3-hydroxy-1,4-naphthoquinone (566C80). Biochem Pharmacol..

[54] Paes LS, Mantilla BS, Barison MJ, Wrenger C, Silber AM (2011). The uniqueness of the *Trypanosoma cruzi* mitochondrion: opportunities to target new drugs against Chagas disease. Curr Pharm Des..

[55] Go RE, Hwang KA, Choi KC (2015). Cytochrome P450 1 family and cancers. J Steroid Biochem Mol Biol..

[56] Steller H (1995). Mechanisms and genes of cellular suicide. Science..

[57] Harder A (2016). The biochemistry of *Haemonchus contortus* and other parasitic nematodes. Adv Parasitol..

[58] Van Gilst MR, Hadjivassiliou H, Jolly A, Yamamoto KR (2005). Nuclear hormone receptor NHR-49 controls fat consumption and fatty acid composition in *C. elegans*. PLoS Biol..

[59] Ratnappan R, Amrit FRG, Chen SW, Gill H, Holden K, Ward J (2014). Germline signals deploy NHR-49 to modulate fatty-acid β-oxidation and desaturation in somatic tissues of *Caenorhabditis elegans*. PLoS Genet..

[60] Nelson DL, Cox MM (2012). Lehninger principles of biochemistry.

[61] Hunt-Newbury R, Viveiros R, Johnsen R, Mah A, Anastas D, Fang L (2007). High-throughput *in vivo* analysis of gene expression in *Caenorhabditis elegans*. PLoS Biol..

[62] Van Gilst MR, Hadjivassiliou H, Yamamoto KR (2005). A *Caenorhabditis elegans* nutrient response system partially dependent on nuclear receptor NHR-49. Proc Natl Acad Sci USA.

[63] Lee DL (2002). The biology of nematodes.

[64] Mullaney BC, Ashrafi K (2009). *Caenorhabditis elegans* fat storage and metabolic regulation. Biochim Biophys Acta..

[65] Lemieux GA, Ashrafi K (2015). Insights and challenges in using *Caenorhabditis elegans* for investigation of fat metabolism. Crit Rev Biochem Mol Biol.

[66] McGhee JD (1987). Purification and characterization of a carboxylesterase from the intestine of the nematode *Caenorhabditis elegans*. Biochemistry..

[67] Kniazeva M, Crawford QT, Seiber M, Wang CY, Han M (2004). Monomethyl branched-chain fatty acids play an essential role in *Caenorhabditis elegans* development. PLoS Biol..

[68] Brock TJ, Browse J, Watts JL (2006). Genetic regulation of unsaturated fatty acid composition in *C. elegans*. PLoS Genet..

[69] Laing ST, Ivens A, Butler V, Ravikumar SP, Laing R, Woods DJ, Gilleard JS (2012). The transcriptional response of *Caenorhabditis elegans* to ivermectin exposure identifies novel genes involved in the response to reduced food intake. PLoS ONE..

