# Supplementary material for: Transcriptional alterations in Caenorhabditis elegans following exposure to an anthelmintic fraction of the plant Picria fel-terrae Lour
Source: Parasit Vectors. 2019 Apr 25;12:181. doi: 10.1186/s13071-019-3429-4 (PMC6485125; doi:10.1186/s13071-019-3429-4)
Supplement: Supplementary file 1 — Additional file 1: Table S1. Summary of sequence libraries and RNAseq data aligned to the C. elegans mRNA. [file 13071_2019_3429_MOESM1_ESM.docx]

Additional file 1: Table S1. Sequence libraries and RNAseq data aligned to the *C. elegans* mRNA

| **Library** | **Total reads** | **Total read pairs** | **Total high-quality reads** | **High quality read pairs** | **Unpaired high-quality reads** | **Aligned pairs reads** | **Aligned pairs reads (%)** |
| --- | --- | --- | --- | --- | --- | --- | --- |
| *C. elegans* exposed fraction 5 Rep 1 | 25,013,948 | 12,506,974 | 22,454,552 | 11,227,276 | 1,074,045 | 20,675,828 | 92.08 |
| *C. elegans* exposed fraction 5 Rep 2 | 24,710,456 | 12,355,228 | 22,168,118 | 11,084,059 | 1,076,388 | 20,342,956 | 91.77 |
| *C. elegans* M9 control Rep 1 | 24,965,096 | 12,482,548 | 22,343,344 | 11,171,672 | 1,098,798 | 20,652,788 | 92.43 |
| *C. elegans* M9 control Rep 2 | 25,042,838 | 12,521,419 | 22,485,372 | 11,242,686 | 1,072,763 | 20,763,256 | 92.34 |
