# Supplementary figures and images for: Transcriptional alterations in Caenorhabditis elegans following exposure to an anthelmintic fraction of the plant Picria fel-terrae Lour
Source: Parasit Vectors. 2019 Apr 25;12:181. doi: 10.1186/s13071-019-3429-4 (PMC6485125; doi:10.1186/s13071-019-3429-4)

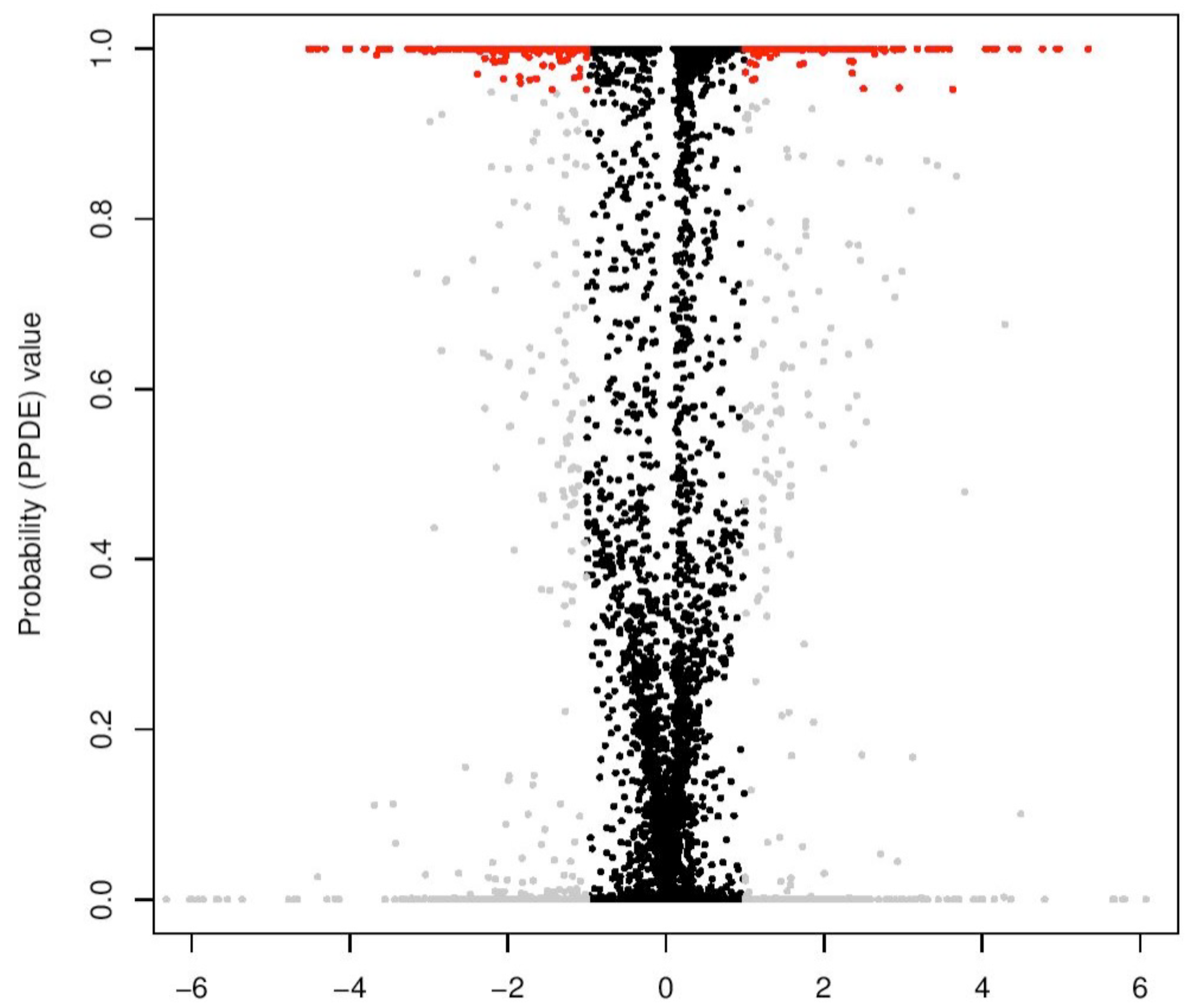

Supplement: Supplementary file 2 — Additional file 2: Figure S1. Differential transcription of protein-encoding genes in C. elegans upon exposure to an anthelmintic fraction of the plant Picria fel-terrae Lour. compared with untreated C. elegans. Genes significantly differentially transcribed at > 2-fold change and a posterior probability (PP) of > 0.95 (red dots); at > 2-fold change and a PP of < 0.95 (grey dots); at < 2-fold change and a PP of < 0.95 (black dots). [file 13071_2019_3429_MOESM2_ESM.tif]
